# Supplementary material for: Exome-wide age-of-onset analysis reveals exonic variants in ERN1 and SPPL2C associated with Alzheimer’s disease
Source: Transl Psychiatry. 2021 Feb 26;11:146. doi: 10.1038/s41398-021-01263-4 (PMC7910483; doi:10.1038/s41398-021-01263-4)
Supplement: Supplementary file 1 — Legends for supplementary figures and tables [file 41398_2021_1263_MOESM1_ESM.docx]

# **Legends for the supplementary materials**

# **Supplementary Text**

Text S1. Additional acknowledgments for ADSP, ADNI, GTEx, and CHS, and a full list of members of the GTEx consortium and their affiliations.

# **Supplementary Tables**

Table S1. Basic characteristics of the six study samples (ADSP, LOADFS, ROSMAP, CHS, GenADA, the ADSP extension study, and ADNI) included in the discovery and replication phases of the age-of-onset association analyses of AD.

Table S2. Summary statistics of the three age-of-onset association analyses of AD using the ADSP sample. Variants with missing rate >2% or MAC ≤10 in each of the analyses were not included in the tables. Model 1: a model with all subjects adjusted for three significant PCs and sex; Model 2: a model with all subjects adjusted for the copy of *APOE* *ε4*, three significant PCs and sex; Model 3: a model with *APOE* *ε4* non-carriers adjusted for three significant PCs and sex. The positions of the SNPs are based on hg19.

Table S3. Summary statistics of the differential DNA methylation analyses of 11 probes in the *ERN1* region using a ROSMAP sample for rs56201815. Beta: the effect size in M-value with respect to a copy of rs56201815-G.

Table S4. Summary statistics of the differential analyses of nine H3K9ac peaks in the *ERN1* region (**±**200k flanking region of rs56201815) using a ROSMAP sample for rs56201815. logFC: log(fold-change) with respect to a copy of rs56201815-G. logCPM: log(count per million) of the peak. LR: likelihood ratio test statistics. Classification: functional annotation of the peak. Median Count: median count of the reads in the peak across the subjects.

Table S5. Results of the cell type-specific eQTL analysis of rs12373123 in six major brain cell types (excitatory neurons, inhibitory neurons, astrocytes, microglia, oligodendrocytes, and OPCs) using the ROSMAP snRNA-seq data in the frontal cortex. logFC: log(fold-change) with respect to a copy of rs12373123-C. logCPM: log(count per million) of the gene expression. LR: likelihood ratio test statistics.

Table S6. Frequency across 24 cohorts among 10,913 subjects included in the ADSP sample.

# **Supplementary Figures**

Figure S1. Q-Q plots of the p-values from the exome-wide age-of-onset association analyses of AD using A) a model with all subjects adjusted for three top significant PCs and sex; B) a model with all subjects adjusted for the copies of *APOE* *ε4*, three top significant PCs and sex; C) a model with *APOE* *ε4* non-carriers adjusted for three top significant PCs and sex. λ: genomic inflation factor.

Figure S2. Normalized expression of *ERN1* between rs56201815-G carriers and non-carriers in A) ten tissues from the GTEx RNA-seq samples; B) peripheral blood from the ADNI microarray sample. In the GTEx samples, there is one rs56201815-G carrier in all tissues except for the colon, in which there are two carriers.

Figure S3. Normalized expression of nine genes (*ARL17A*, *KANSL1*, *LRRC37A3*, *CRHR1*, *SPPL2C*, *LRRC37A*, *LRRC37A2, PLEKHM1*, *ARHGAP27*) near rs12373123 of 44 subjects in six major brain cell types (astrocytes, excitatory neurons, inhibitory neurons, microglia, OPCs, and oligodendrocytes). All cells from the same subject were first pooled within each cell type, and the raw counts were aggregated. The normalized gene expression was adjusted for age, sex, and AD status. Expression of *SPPL2C* was observed only in neuronal cells.

Figure S4. Normalized expression of *ERN1* of 39 WGS subjects (including one rs56201815-G carrier) in astrocytes, excitatory neurons, and oligodendrocytes. All cells from the same subject were first pooled within each cell type, and the raw counts were aggregated. The normalized gene expression was adjusted for age, sex, and AD status.

Figure S5. P-values of the gene-based association analyses of age-of-onset of AD using summary statistics based on (A) Model 2: a model with all subjects in the ADSP project adjusted for the copies of *APOE* *ε4*; (B) Model 3: a model with only *APOE* *ε4* non-carriers. Top genes with a p-value <1e-04 were highlighted. The red horizontal line is a p-value threshold based on the Bonferroni correction (0.05/17,000=3e-06).
