# Supplementary material for: Exome-wide age-of-onset analysis reveals exonic variants in ERN1 and SPPL2C associated with Alzheimer’s disease
Source: Transl Psychiatry. 2021 Feb 26;11:146. doi: 10.1038/s41398-021-01263-4 (PMC7910483; doi:10.1038/s41398-021-01263-4)
Supplement: Supplementary file 2 — Supplementary Text S1 [file 41398_2021_1263_MOESM2_ESM.docx]

Supplementary materials Text S1. for “Exome-wide age-of-onset analysis reveals exonic variants in *ERN1*, *TACR3*, and *SPPL2C* associated with Alzheimer’s disease”

# **Acknowledgments**

## ADSP

The Alzheimer’s Disease Sequencing Project (ADSP) is comprised of two Alzheimer’s Disease (AD) genetics consortia and three National Human Genome Research Institute (NHGRI) funded Large Scale Sequencing and Analysis Centers (LSAC). The two AD genetics consortia are the Alzheimer’s Disease Genetics Consortium (ADGC) funded by NIA (U01 AG032984), and the Cohorts for Heart and Aging Research in Genomic Epidemiology (CHARGE) funded by NIA (R01 AG033193), the National Heart, Lung, and Blood Institute (NHLBI), other National Institute of Health (NIH) institutes and other foreign governmental and non-governmental organizations. The Discovery Phase analysis of sequence data is supported through UF1AG047133 (to Drs. Schellenberg, Farrer, Pericak-Vance, Mayeux, and Haines); U01AG049505 to Dr. Seshadri; U01AG049506 to Dr. Boerwinkle; U01AG049507 to Dr. Wijsman; and U01AG049508 to Dr. Goate and the Discovery Extension Phase analysis is supported through U01AG052411 to Dr. Goate, U01AG052410 to Dr. Pericak-Vance and U01 AG052409 to Drs. Seshadri and Fornage. Data generation and harmonization in the Follow-up Phases is supported by U54AG052427 (to Drs. Schellenberg and Wang).

The ADGC cohorts include: Adult Changes in Thought (ACT), the Alzheimer’s Disease Centers (ADC), the Chicago Health and Aging Project (CHAP), the Memory and Aging Project (MAP), Mayo Clinic (MAYO), Mayo Parkinson’s Disease controls, University of Miami, the Multi-Institutional Research in Alzheimer’s Genetic Epidemiology Study (MIRAGE), the National Cell Repository for Alzheimer’s Disease (NCRAD), the National Institute on Aging Late Onset Alzheimer's Disease Family Study (NIA-LOAD), the Religious Orders Study (ROS), the Texas Alzheimer’s Research and Care Consortium (TARC), Vanderbilt University/Case Western Reserve University (VAN/CWRU), the Washington Heights-Inwood Columbia Aging Project (WHICAP) and the Washington University Sequencing Project (WUSP), the Columbia University Hispanic- Estudio Familiar de Influencia Genetica de Alzheimer (EFIGA), the University of Toronto (UT), and Genetic Differences (GD).

The CHARGE cohorts are supported in part by National Heart, Lung, and Blood Institute (NHLBI) infrastructure grant HL105756 (Psaty), RC2HL102419 (Boerwinkle) and the neurology working group is supported by the National Institute on Aging (NIA) R01 grant AG033193. The CHARGE cohorts participating in the ADSP include the following: Austrian Stroke Prevention Study (ASPS), ASPS-Family study, and the Prospective Dementia Registry-Austria (ASPS/PRODEM-Aus), the Atherosclerosis Risk in Communities (ARIC) Study, the Cardiovascular Health Study (CHS), the Erasmus Rucphen Family Study (ERF), the Framingham Heart Study (FHS), and the Rotterdam Study (RS). ASPS is funded by the Austrian Science Fond (FWF) grant number P20545-P05 and P13180 and the Medical University of Graz. The ASPS-Fam is funded by the Austrian Science Fund (FWF) project I904),the EU Joint Programme - Neurodegenerative Disease Research (JPND) in frame of the BRIDGET project (Austria, Ministry of Science) and the Medical University of Graz and the Steiermärkische Krankenanstalten Gesellschaft. PRODEM-Austria is supported by the Austrian Research Promotion agency (FFG) (Project No. 827462) and by the Austrian National Bank (Anniversary Fund, project 15435. ARIC research is carried out as a collaborative study supported by NHLBI contracts (HHSN268201100005C, HHSN268201100006C, HHSN268201100007C, HHSN268201100008C, HHSN268201100009C, HHSN268201100010C, HHSN268201100011C, and HHSN268201100012C). Neurocognitive data in ARIC is collected by U01 2U01HL096812, 2U01HL096814, 2U01HL096899, 2U01HL096902, 2U01HL096917 from the NIH (NHLBI, NINDS, NIA and NIDCD), and with previous brain MRI examinations funded by R01-HL70825 from the NHLBI. CHS research was supported by contracts HHSN268201200036C, HHSN268200800007C, N01HC55222, N01HC85079, N01HC85080, N01HC85081, N01HC85082, N01HC85083, N01HC85086, and grants U01HL080295 and U01HL130114 from the NHLBI with additional contribution from the National Institute of Neurological Disorders and Stroke (NINDS). Additional support was provided by R01AG023629, R01AG15928, and R01AG20098 from the NIA. FHS research is supported by NHLBI contracts N01-HC-25195 and HHSN268201500001I. This study was also supported by additional grants from the NIA (R01s AG054076, AG049607 and AG033040 and NINDS (R01 NS017950). The ERF study as a part of EUROSPAN (European Special Populations Research Network) was supported by European Commission FP6 STRP grant number 018947 (LSHG-CT-2006-01947) and also received funding from the European Community's Seventh Framework Programme (FP7/2007-2013)/grant agreement HEALTH-F4-2007-201413 by the European Commission under the programme "Quality of Life and Management of the Living Resources" of 5th Framework Programme (no. QLG2-CT-2002-01254). High-throughput analysis of the ERF data was supported by a joint grant from the Netherlands Organization for Scientific Research and the Russian Foundation for Basic Research (NWO-RFBR 047.017.043). The Rotterdam Study is funded by Erasmus Medical Center and Erasmus University, Rotterdam, the Netherlands Organization for Health Research and Development (ZonMw), the Research Institute for Diseases in the Elderly (RIDE), the Ministry of Education, Culture and Science, the Ministry for Health, Welfare and Sports, the European Commission (DG XII), and the municipality of Rotterdam. Genetic data sets are also supported by the Netherlands Organization of Scientific Research NWO Investments (175.010.2005.011, 911-03-012), the Genetic Laboratory of the Department of Internal Medicine, Erasmus MC, the Research Institute for Diseases in the Elderly (014-93-015; RIDE2), and the Netherlands Genomics Initiative (NGI)/Netherlands Organization for Scientific Research (NWO) Netherlands Consortium for Healthy Aging (NCHA), project 050-060-810. All studies are grateful to their participants, faculty and staff. The content of these manuscripts is solely the responsibility of the authors and does not necessarily represent the official views of the National Institutes of Health or the U.S. Department of Health and Human Services.

The four LSACs are: the Human Genome Sequencing Center at the Baylor College of Medicine (U54 HG003273), the Broad Institute Genome Center (U54HG003067), The American Genome Center at the Uniformed Services University of the Health Sciences (U01AG057659), and the Washington University Genome Institute (U54HG003079).

Biological samples and associated phenotypic data used in primary data analyses were stored at Study Investigators institutions, and at the National Cell Repository for Alzheimer’s Disease (NCRAD, U24AG021886) at Indiana University funded by NIA. Associated Phenotypic Data used in primary and secondary data analyses were provided by Study Investigators, the NIA funded Alzheimer’s Disease Centers (ADCs), and the National Alzheimer’s Coordinating Center (NACC, U01AG016976) and the National Institute on Aging Genetics of Alzheimer’s Disease Data Storage Site (NIAGADS, U24AG041689) at the University of Pennsylvania, funded by NIA, and at the Database for Genotypes and Phenotypes (dbGaP) funded by NIH. This research was supported in part by the Intramural Research Program of the National Institutes of health, National Library of Medicine. Contributors to the Genetic Analysis Data included Study Investigators on projects that were individually funded by NIA, and other NIH institutes, and by private U.S. organizations, or foreign governmental or nongovernmental organizations.

## GTEx

The Genotype-Tissue Expression (GTEx) Project was supported by the Common Fund of the Office of the Director of the National Institutes of Health, and by NCI, NHGRI, NHLBI, NIDA, NIMH, and NINDS.

## ADNI

Data collection and sharing for this project was funded by the Alzheimer's Disease Neuroimaging Initiative (ADNI) (National Institutes of Health Grant U01 AG024904) and DOD ADNI (Department of Defense award number W81XWH-12-2-0012). ADNI is funded by the National Institute on Aging, the National Institute of Biomedical Imaging and Bioengineering, and through generous contributions from the following: AbbVie, Alzheimer’s Association; Alzheimer’s Drug Discovery Foundation; Araclon Biotech; BioClinica, Inc.; Biogen; Bristol-Myers Squibb Company; CereSpir, Inc.; Cogstate; Eisai Inc.; ElanPharmaceuticals, Inc.; Eli Lilly and Company; EuroImmun; F. Hoffmann-La Roche Ltd and its affiliated company Genentech, Inc.; Fujirebio; GE Healthcare; IXICO Ltd.; Janssen Alzheimer Immunotherapy Research & Development, LLC.; Johnson & Johnson Pharmaceutical Research & Development LLC.; Lumosity; Lundbeck; Merck & Co., Inc.; Meso Scale Diagnostics, LLC.; NeuroRx Research; Neurotrack Technologies; Novartis Pharmaceuticals Corporation; Pfizer Inc.; Piramal Imaging; Servier; Takeda Pharmaceutical Company; and Transition Therapeutics. The Canadian Institutes of Health Research is providing funds to support ADNI clinical sites in Canada. Private sector contributions are facilitated by the Foundation for the National Institutes of Health (www.fnih.org). The grantee organization is the Northern California Institute for Research and Education, and the study is coordinated by the Alzheimer’s Therapeutic Research Institute at the University of Southern California. ADNI data are disseminated by the Laboratory for NeuroImaging at the University of SouthernCalifornia.

## CHS

The Cardiovascular Health Study (CHS) was supported by contract numbers N01-HC-85079, N01-HC-85080, N01-HC-85081, N01-HC-85082, N01-HC-85083, N01-HC- 85084, N01-HC-85085, N01-HC-85086, N01-HC-35129, N01 HC-15103, N01 HC-55222, N01-HC-75150, N01-HC-45133, N01-HC-85239, and HHSN268201200036C; grant numbers U01 HL080295 from the NHLBI and R01 AG-023629 from the NIA, with additional contribution from the National Institute of Neurological Disorders and Stroke. A full list of principal CHS investigators and institutions can be found at http://chs-nhlbi.org. This manuscript was not prepared in collaboration with CHS investigators and does not necessarily reflect the opinions or views of CHS, or the NHLBI. Support for the genotyping through the CARe Study was provided by NHLBI Contract N01-HC-65226.

# GTEx Consortium^†^

Laboratory and Data Analysis Coordinating Center (LDACC): François Aguet^1^, Shankara Anand^1^, Kristin G. Ardlie^1^, Stacey Gabriel^1^, Gad Getz^1,30,31^, Aaron Graubert^1^, Kane Hadley^1^, Robert E. Handsaker^33,34,35^, Katherine H. Huang^1^, Seva Kashin^33,34,35^, Xiao Li^1^, Daniel G. MacArthur^34,36^, Samuel R. Meier^1^, Jared L. Nedzel^1^, Duyen T. Nguyen^1^, Ayellet V. Segrè^1,17^, Ellen Todres^1^

Analysis Working Group Funded by GTEx Project Grants: François Aguet^1^, Shankara Anand^1^, Kristin G. Ardlie^1^, Brunilda Balliu^41^, Alvaro N. Barbeira^2^, Alexis Battle^18,11^, Rodrigo Bonazzola^2^, Andrew Brown^3,4^, Christopher D. Brown^24^, Stephane E. Castel^5,6^, Donald F. Conrad^42,43^, Daniel J. Cotter^29^, Nancy Cox^16^, Sayantan Das^26^, Olivia M. deGoede^29^, Emmanouil T. Dermitzakis^3,27,28^, Jonah Einson^44,5^, Barbara E. Engelhardt^7,8^, Eleazar Eskin^45^, Tiffany Y. Eulalio^46^, Nicole M. Ferraro^46^, Elise D. Flynn^5,6^, Laure Fresard^12^, Eric R. Gamazon^13,14,15,16^, Diego Garrido-Martín^22^, Nicole R. Gay^29^, Gad A. Getz^1,30,31^, Michael J. Gloudemans^46^, Aaron Graubert^1^, Roderic Guigó^22,32^, Kane Hadley^1^, Andrew R. Hamel^17,1^, Robert E. Handsaker^33,34,35^, Yuan He^18^, Paul J. Hoffman^5^, Farhad Hormozdiari^19,1^, Lei Hou^47,1^, Katherine H. Huang^1^, Hae Kyung Im^2^, Brian Jo^7,8^, Silva Kasela^5,6^, Seva Kashin^33,34,35^, Manolis Kellis^47,1^, Sarah Kim-Hellmuth^5,6,9^, Alan Kwong^26^, Tuuli Lappalainen^5,6^, Xiao Li^1^, Xin Li^12^, Yanyu Liang^2^, Daniel G. MacArthur^34,36^, Serghei Mangul^45,48^, Samuel R. Meier^1^, Pejman Mohammadi^5,6,20,21^, Stephen B. Montgomery^12,29^, Manuel Muñoz-Aguirre^22,23^, Daniel C. Nachun^12^, Jared L. Nedzel^1^, Duyen T. Nguyen^1^, Andrew B. Nobel^49^, Meritxell Oliva^2,10^, YoSon Park^24,25^, Yongjin Park^47,1^, Princy Parsana^11^, Abhiram S. Rao^50^, Ferran Reverter^51^, John M. Rouhana^17,1^, Chiara Sabatti^52^, Ashis Saha^11^, Ayellet V. Segrè^1,17^, Andrew D. Skol^2,53^, Matthew Stephens^37^, Barbara E. Stranger^2,38^, Benjamin J. Strober^18^, Nicole A. Teran^12^, Ellen Todres^1^, Ana Viñuela^39,3,27,28^, Gao Wang^37^, Xiaoquan Wen^26^, Fred Wright^54^, Valentin Wucher^22^, Yuxin Zou^40^

Analysis Working Group Not Funded by GTEx Project Grants: Pedro G. Ferreira^55,56,57,58^, Gen Li^59^, Marta Melé^60^, Esti Yeger-Lotem^61,62^

Leidos Biomedical Project Management: Mary E. Barcus^63^, Debra Bradbury^63^, Tanya Krubit^63^, Jeffrey A. McLean^63^, Liqun Qi^63^, Karna Robinson^63^, Nancy V. Roche^63^, Anna M. Smith^63^, Leslie Sobin^63^, David E. Tabor^63^, Anita Undale^63^

Biospecimen Collection Source Sites: Jason Bridge^64^, Lori E. Brigham^65^, Barbara A. Foster^66^, Bryan M. Gillard^66^, Richard Hasz^67^, Marcus Hunter^68^, Christopher Johns^69^, Mark Johnson^70^, Ellen Karasik^66^, Gene Kopen^71^, William F. Leinweber^71^, Alisa McDonald^71^, Michael T. Moser^66^, Kevin Myer^68^, Kimberley D. Ramsey^66^, Brian Roe^68^, Saboor Shad^71^, Jeffrey A. Thomas^71,70^, Gary Walters^70^, Michael Washington^70^, Joseph Wheeler^69^

Biospecimen Core Resource: Scott D. Jewell^72^, Daniel C. Rohrer^72^, Dana R. Valley^72^

Brain Bank Repository: David A. Davis^73^, Deborah C. Mash^73^

Pathology: Mary E. Barcus^63^, Philip A. Branton^74^, Leslie Sobin^63^

ELSI Study: Laura K. Barker^75^, Heather M. Gardiner^75^, Maghboeba Mosavel^76^, Laura A. Siminoff^75^

Genome Browser Data Integration and Visualization: Paul Flicek^77^, Maximilian Haeussler^78^, Thomas Juettemann^77^, W. James Kent^78^, Christopher M. Lee^78^, Conner C. Powell^78^, Kate R. Rosenbloom^78^, Magali Ruffier^77^, Dan Sheppard^77^, Kieron Taylor^77^, Stephen J. Trevanion^77^, Daniel R. Zerbino^77^

eGTEx Groups: Nathan S. Abell^29^, Joshua Akey^79^, Lin Chen^10^, Kathryn Demanelis^10^, Jennifer A. Doherty^80^, Andrew P. Feinberg^81^, Kasper D. Hansen^82^, Peter F. Hickey^83^, Lei Hou^47,1^, Farzana Jasmine^10^, Lihua Jiang^29^, Rajinder Kaul^84,85^, Manolis Kellis^47,1^, Muhammad G. Kibriya^10^, Jin Billy Li^29^, Qin Li^29^, Shin Lin^86^, Sandra E. Linder^29^, Stephen B. Montgomery^12,29^, Meritxell Oliva^2,10^, Yongjin Park^47,1^, Brandon L. Pierce^10^, Lindsay F. Rizzardi^87^, Andrew D. Skol^2,53^, Kevin S. Smith^12^, Michael Snyder^29^, John Stamatoyannopoulos^84,88^, Barbara E. Stranger^2,38^, Hua Tang^29^, Meng Wang^29^

NIH Program Management: Philip A. Branton^74^, Latarsha J. Carithers^74,89^, Ping Guan^74^, Susan E. Koester^90^, A. Roger Little^91^, Helen M. Moore^74^, Concepcion R. Nierras^92^, Abhi K. Rao^74^, Jimmie B. Vaught^74^, Simona Volpi^93^

^1^The Broad Institute of MIT and Harvard, Cambridge, MA, USA. ^2^Section of Genetic Medicine, Department of Medicine, University of Chicago, Chicago, IL, USA. ^3^Department of Genetic Medicine and Development, University of Geneva Medical School, Geneva, Switzerland. ^4^Population Health and Genomics, University of Dundee, Dundee, Scotland, UK. ^5^New York Genome Center, New York, NY, USA. ^6^Department of Systems Biology, Columbia University, New York, NY, USA. ^7^Department of Computer Science, Princeton University, Princeton, NJ, USA. ^8^Center for Statistics and Machine Learning, Princeton University, Princeton, NJ, USA. ^9^Statistical Genetics, Max Planck Institute of Psychiatry, Munich, Germany. ^10^Department of Public Health Sciences, University of Chicago, Chicago, IL, USA. ^11^Department of Computer Science, Johns Hopkins University, Baltimore, MD, USA. ^12^Department of Pathology, Stanford University, Stanford, CA, USA. ^13^Data Science Institute, Vanderbilt University, Nashville, TN, USA. ^14^Clare Hall, University of Cambridge, Cambridge, UK. ^15^MRC Epidemiology Unit, University of Cambridge, Cambridge, UK. ^16^Division of Genetic Medicine, Department of Medicine, Vanderbilt University Medical Center, Nashville, TN, USA. ^17^Ocular Genomics Institute, Massachusetts Eye and Ear, Harvard Medical School, Boston, MA, USA. ^18^Department of Biomedical Engineering, Johns Hopkins University, Baltimore, MD, USA. ^19^Department of Epidemiology, Harvard T.H. Chan School of Public Health, Boston, MA, USA. ^20^Scripps Research Translational Institute, La Jolla, CA, USA. ^21^Department of Integrative Structural and Computational Biology, The Scripps Research Institute, La Jolla, CA, USA. ^22^Centre for Genomic Regulation (CRG), The Barcelona Institute for Science and Technology, Barcelona, Catalonia, Spain. ^23^Department of Statistics and Operations Research, Universitat Politècnica de Catalunya (UPC), Barcelona, Catalonia, Spain. ^24^Department of Genetics, University of Pennsylvania, Perelman School of Medicine, Philadelphia, PA, USA. ^25^Department of Systems Pharmacology and Translational Therapeutics, University of Pennsylvania, Perelman School of Medicine, Philadelphia, PA, USA. ^26^Department of Biostatistics, University of Michigan, Ann Arbor, MI, USA.^27^Institute for Genetics and Genomics in Geneva (iGE3), University of Geneva, Geneva, Switzerland. ^28^Swiss Institute of Bioinformatics, Geneva, Switzerland. ^29^Department of Genetics, Stanford University, Stanford, CA, USA. ^30^Cancer Center and Department of Pathology, Massachusetts General Hospital, Boston, MA, USA. ^31^Harvard Medical School, Boston, MA, USA. ^32^Universitat Pompeu Fabra (UPF), Barcelona, Catalonia, Spain. ^33^Department of Genetics, Harvard Medical School, Boston, MA, USA. ^34^Program in Medical and Population Genetics, The Broad Institute of Massachusetts Institute of Technology and Harvard University, Cambridge, MA, USA. ^35^Stanley Center for Psychiatric Research, Broad Institute, Cambridge, MA, USA. ^36^Analytic and Translational Genetics Unit, Massachusetts General Hospital, Boston, MA, USA. ^37^Department of Human Genetics, University of Chicago, Chicago, IL, USA. ^38^Center for Genetic Medicine, Department of Pharmacology, Northwestern University, Feinberg School of Medicine, Chicago, IL, USA. ^39^Department of Twin Research and Genetic Epidemiology, King’s College London, London, UK. ^40^Department of Statistics, University of Chicago, Chicago, IL, USA. ^41^Department of Biomathematics, University of California, Los Angeles, Los Angeles, CA, USA. ^42^Department of Genetics, Washington University School of Medicine, St. Louis, MO, USA. ^43^Division of Genetics, Oregon National Primate Research Center, Oregon Health & Science University, Portland, OR, USA. ^44^Department of Biomedical Informatics, Columbia University, New York, NY, USA. ^45^Department of Computer Science, University of California, Los Angeles, Los Angeles, CA, USA.^46^Program in Biomedical Informatics, Stanford University School of Medicine, Stanford, CA, USA. ^47^Computer Science and Artificial Intelligence Laboratory, Massachusetts Institute of Technology, Cambridge, MA, USA. ^48^Department of Clinical Pharmacy, School of Pharmacy, University of Southern California, Los Angeles, CA, USA. ^49^Department of Statistics and Operations Research and Department of Biostatistics, University of North Carolina, Chapel Hill, NC, USA. ^50^Department of Bioengineering, Stanford University, Stanford, CA, USA. ^51^Department of Genetics, Microbiology and Statistics, University of Barcelona, Barcelona, Spain. ^52^Departments of Biomedical Data Science and Statistics, Stanford University, Stanford, CA, USA. ^53^Department of Pathology and Laboratory Medicine, Ann & Robert H. Lurie Children’s Hospital of Chicago, Chicago, IL, USA. ^54^Bioinformatics Research Center and Departments of Statistics and Biological Sciences, North Carolina State University, Raleigh, NC, USA. ^55^Department of Computer Sciences, Faculty of Sciences, University of Porto, Porto, Portugal. ^56^Instituto de Investigação e Inovação em Saú de, University of Porto, Porto, Portugal. ^57^Institute of Molecular Pathology and Immunology, University of Porto, Porto, Portugal. ^58^Laboratory of Artificial Intelligence and Decision Support, Institute for Systems and Computer Engineering, Technology and Science, Porto, Portugal. ^59^Columbia University Mailman School of Public Health, New York, NY, USA. ^60^Life Sciences Department, Barcelona Supercomputing Center, Barcelona, Spain. ^61^Department of Clinical Biochemistry and Pharmacology, Ben-Gurion University of the Negev, Beer-Sheva, Israel.^62^National Institute for Biotechnology in the Negev, Beer-Sheva, Israel. ^63^Leidos Biomedical, Rockville, MD, USA. ^64^UNYTS, Buffalo, NY, USA. ^65^Washington Regional Transplant Community, Annandale, VA, USA. ^66^Therapeutics, Roswell Park Comprehensive Cancer Center, Buffalo, NY, USA. ^67^Gift of Life Donor Program, Philadelphia, PA, USA. ^68^Life Gift, Houston, TX, USA. ^69^Center for Organ Recovery and Education, Pittsburgh, PA, USA. ^70^LifeNet Health, Virginia Beach, VA, USA. ^71^National Disease Research Interchange, Philadelphia, PA, USA. ^72^Van Andel Research Institute, Grand Rapids, MI, USA. ^73^Department of Neurology, University of Miami Miller School of Medicine, Miami, FL, USA. ^74^Biorepositories and Biospecimen Research Branch, Division of Cancer Treatment and Diagnosis, National Cancer Institute, Bethesda, MD, USA. ^75^College of Public Health, Temple University, Philadelphia, PA, USA. ^76^Virginia Commonwealth University, Richmond, VA, USA. ^77^European Molecular Biology Laboratory, European Bioinformatics Institute, Hinxton, UK. ^78^Genomics Institute, University of California Santa Cruz, Santa Cruz, CA, USA. ^79^Carl Icahn Laboratory, Princeton University, Princeton, NJ, USA. ^80^Department of Population Health Sciences, The University of Utah, Salt Lake City, UT, USA. ^81^Departments of Medicine, Biomedical Engineering, and Mental Health, Johns Hopkins University, Baltimore, MD, USA. ^82^Department of Biostatistics, Bloomberg School of Public Health, Johns Hopkins University, Baltimore, MD, USA. ^83^Department of Medical Biology, The Walter and Eliza Hall Institute of Medical Research, Parkville, Victoria, Australia. ^84^Altius Institute for Biomedical Sciences, Seattle, WA, USA. ^85^Division of Genetics, University of Washington, Seattle, WA, USA. ^86^Department of Cardiology, University of Washington, Seattle, WA, USA. ^87^Hudson Alpha Institute for Biotechnology, Huntsville, AL, USA. ^88^Genome Sciences, University of Washington, Seattle, WA, USA. ^89^National Institute of Dental and Craniofacial Research, Bethesda, MD, USA. ^90^Division of Neuroscience and Basic Behavioral Science, National Institute of Mental Health, National Institutes of Health, Bethesda, MD, USA. ^91^National Institute on Drug Abuse, Bethesda, MD, USA. ^92^Office of Strategic Coordination, Division of Program Coordination, Planning and Strategic Initiatives, Office of the Director, National Institutes of Health, Rockville, MD, USA. ^93^Division of Genomic Medicine, National Human Genome Research Institute, Bethesda, MD, USA.

†Alphabetical order
