# Supplementary figures and images for: Exome-wide age-of-onset analysis reveals exonic variants in ERN1 and SPPL2C associated with Alzheimer’s disease
Source: Transl Psychiatry. 2021 Feb 26;11:146. doi: 10.1038/s41398-021-01263-4 (PMC7910483; doi:10.1038/s41398-021-01263-4)

A)

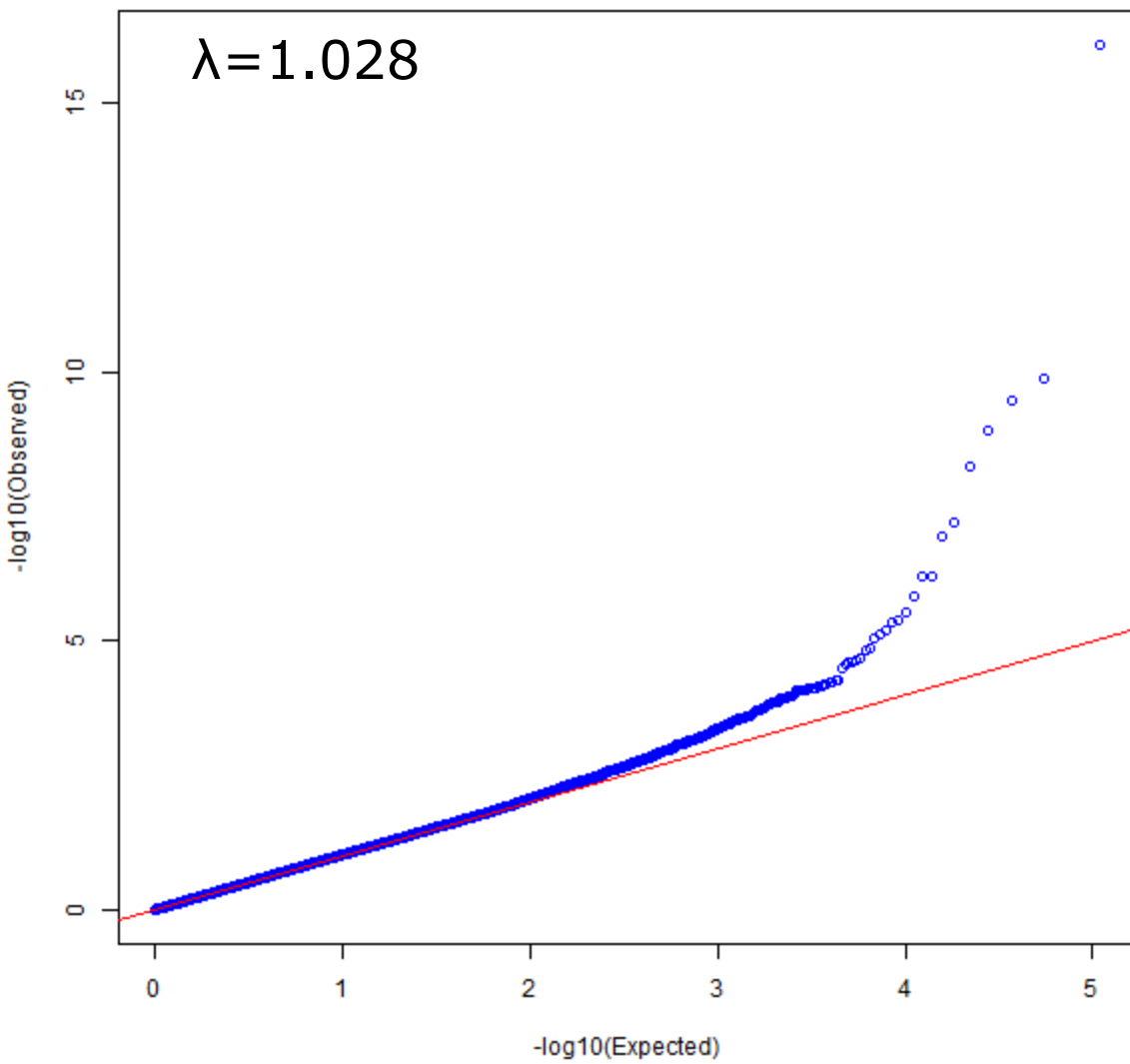

B)

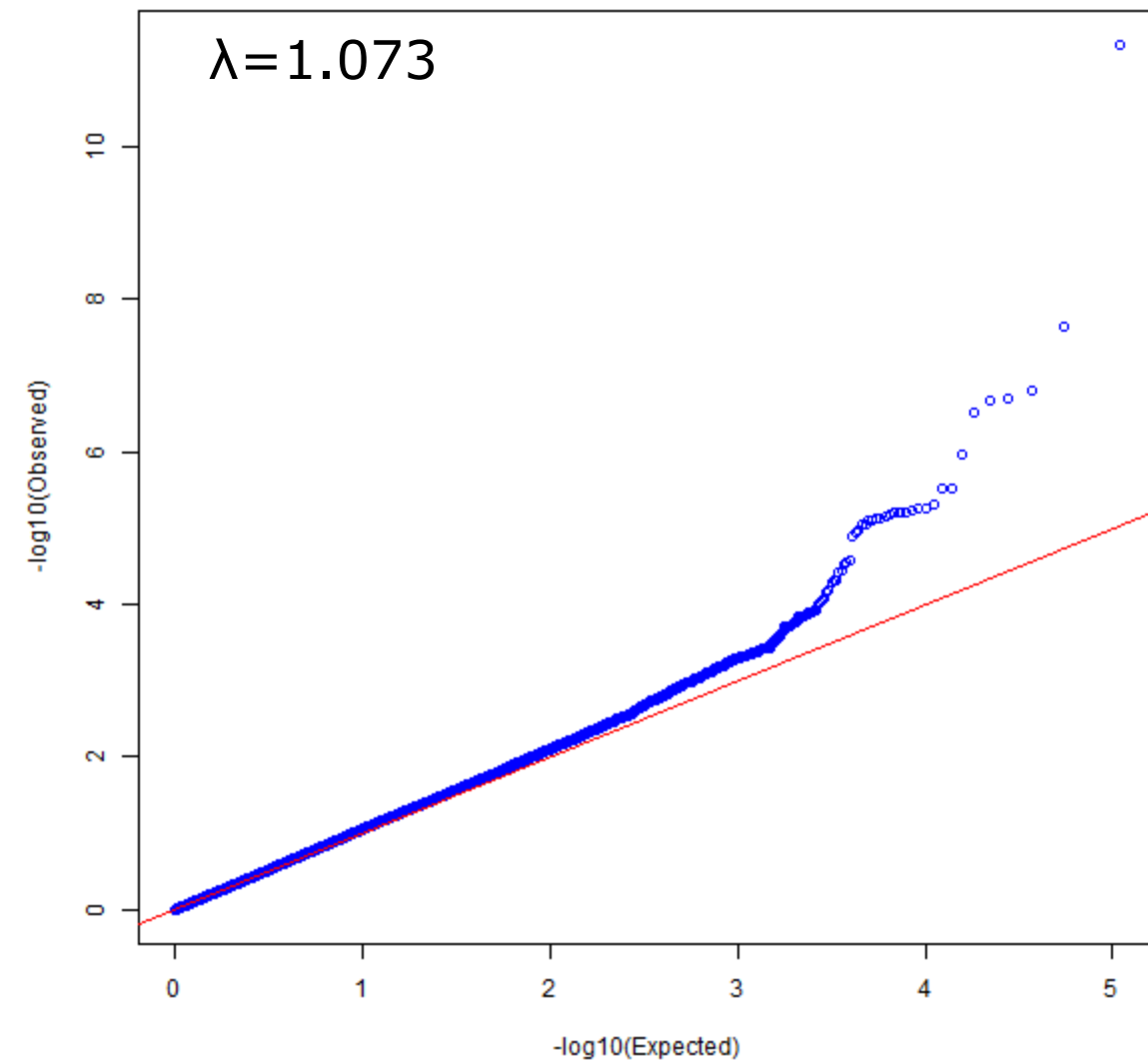

C)

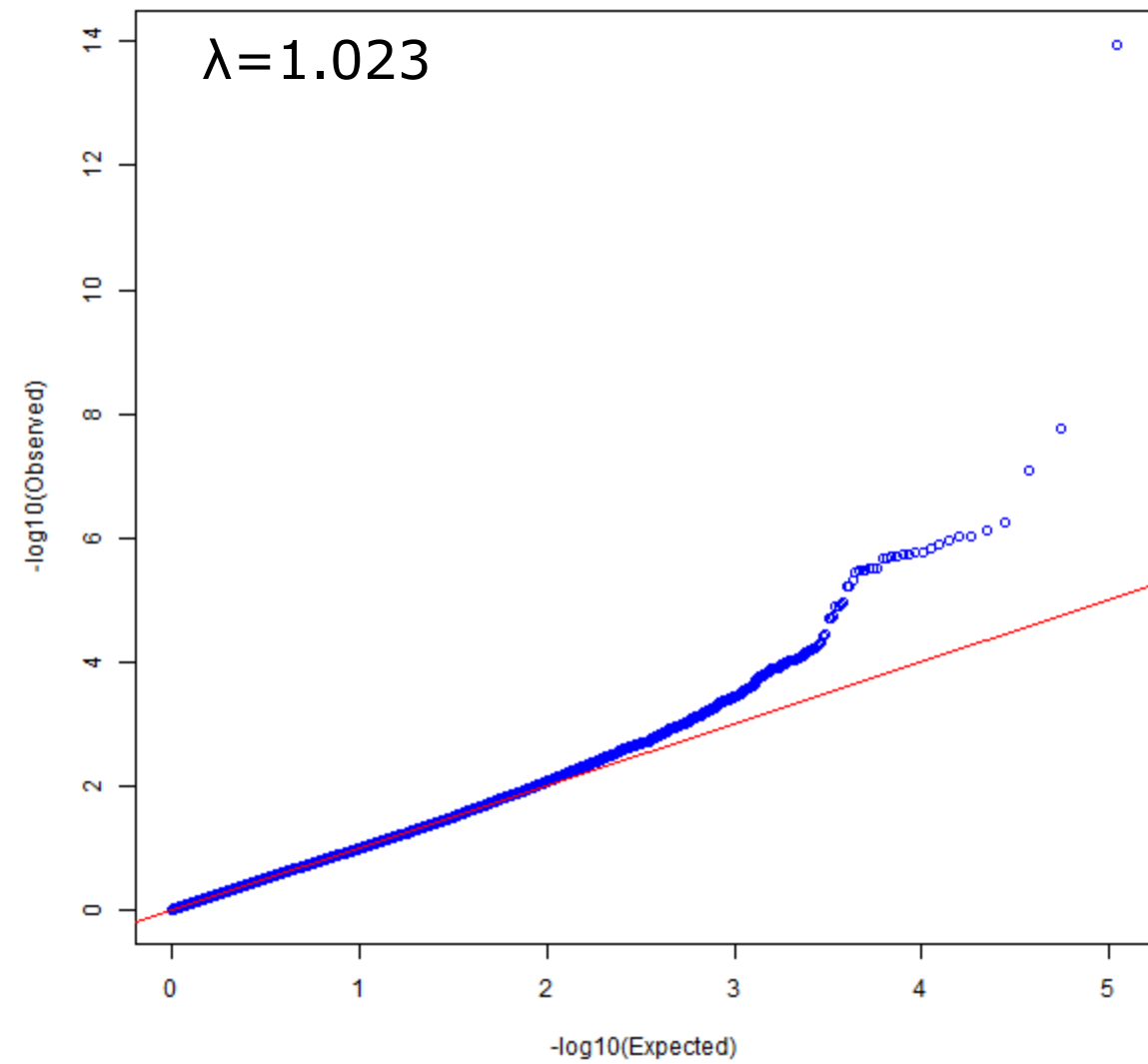

Supplement: Supplementary file 9 — Supplementary Figure S1 [file 41398_2021_1263_MOESM9_ESM.pdf]

A)

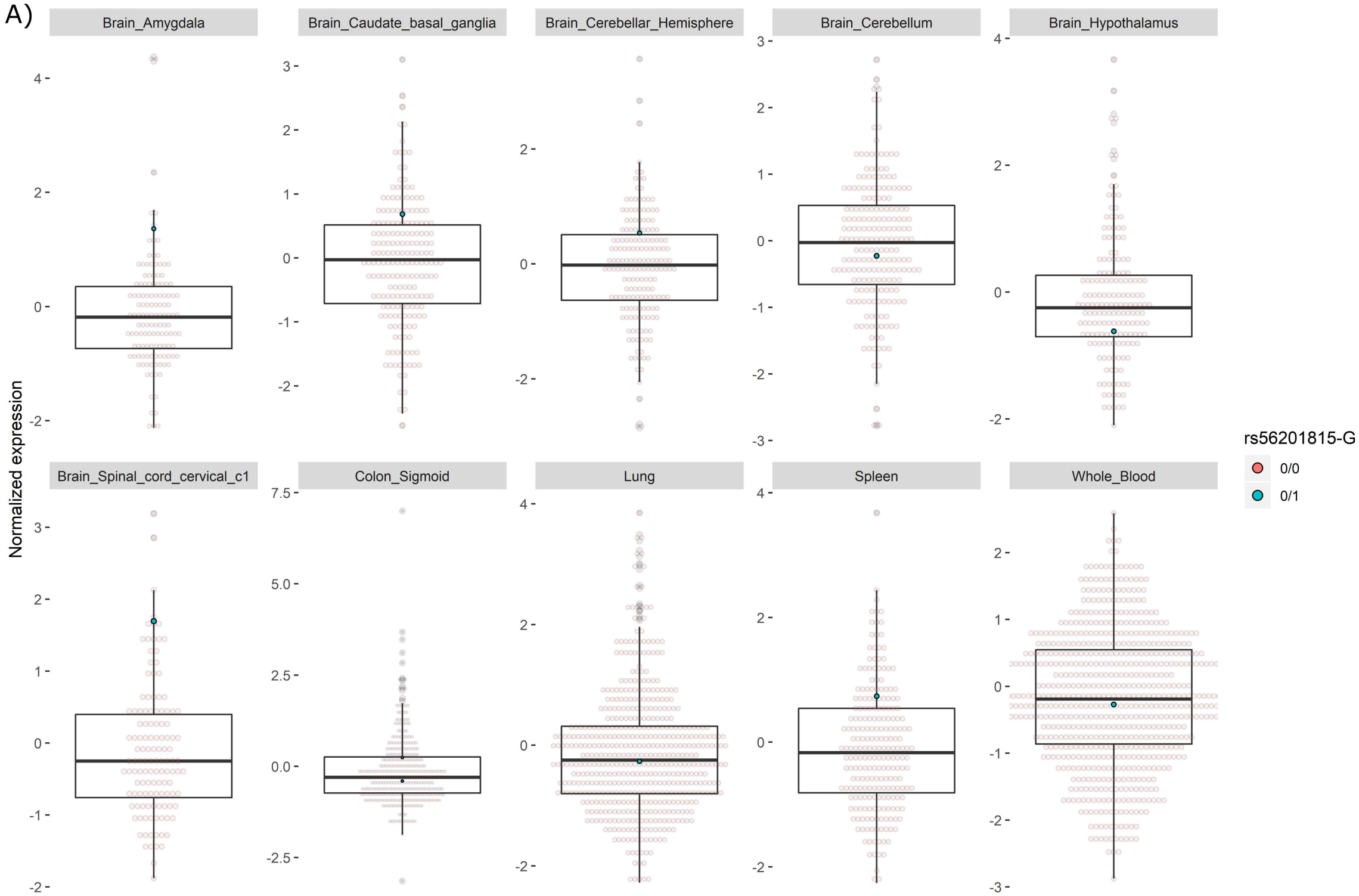

B)

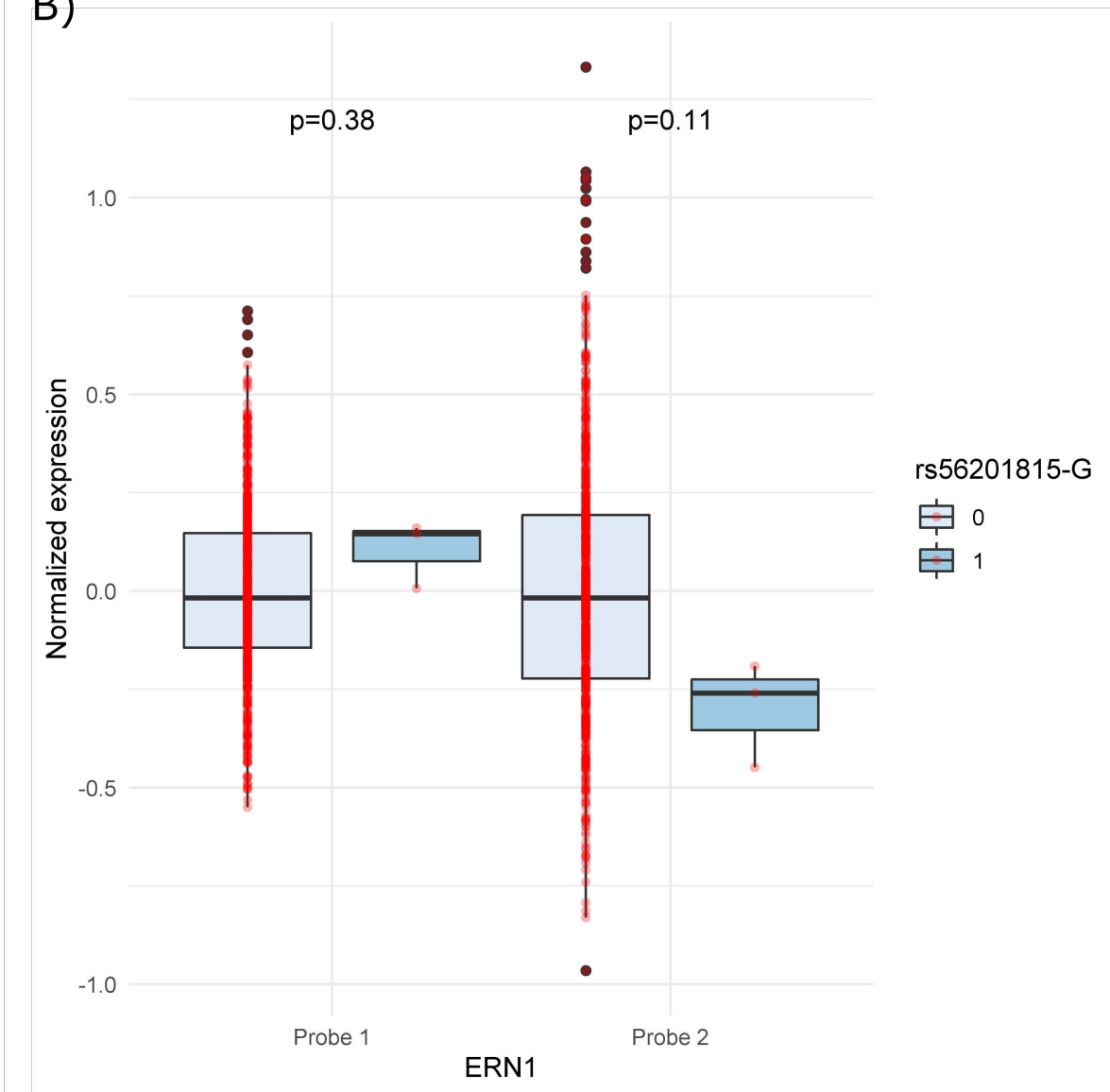

Supplement: Supplementary file 10 — Supplementary Figure S2 [file 41398_2021_1263_MOESM10_ESM.pdf]

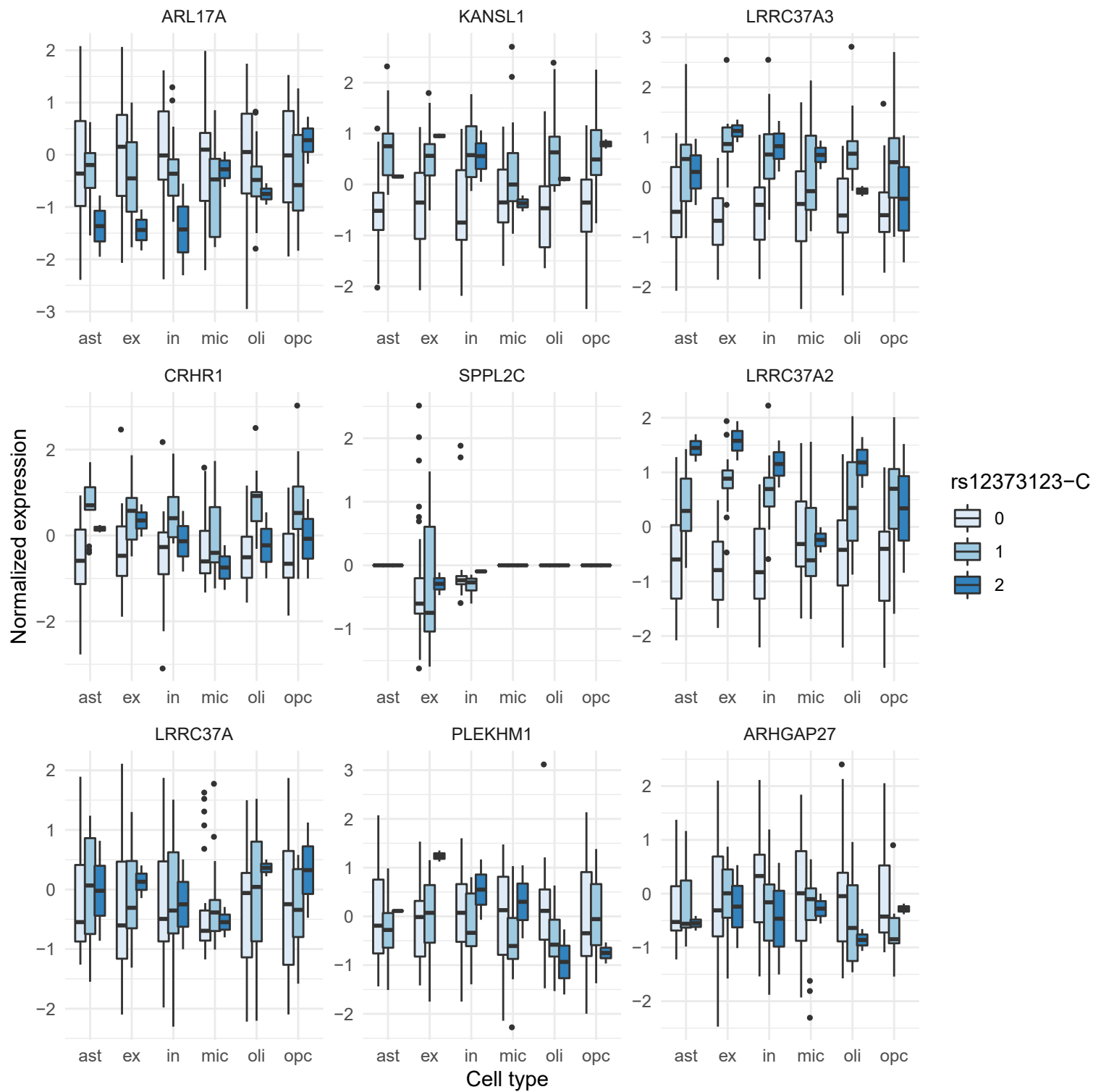

Supplement: Supplementary file 11 — Supplementary Figure S3 [file 41398_2021_1263_MOESM11_ESM.pdf]

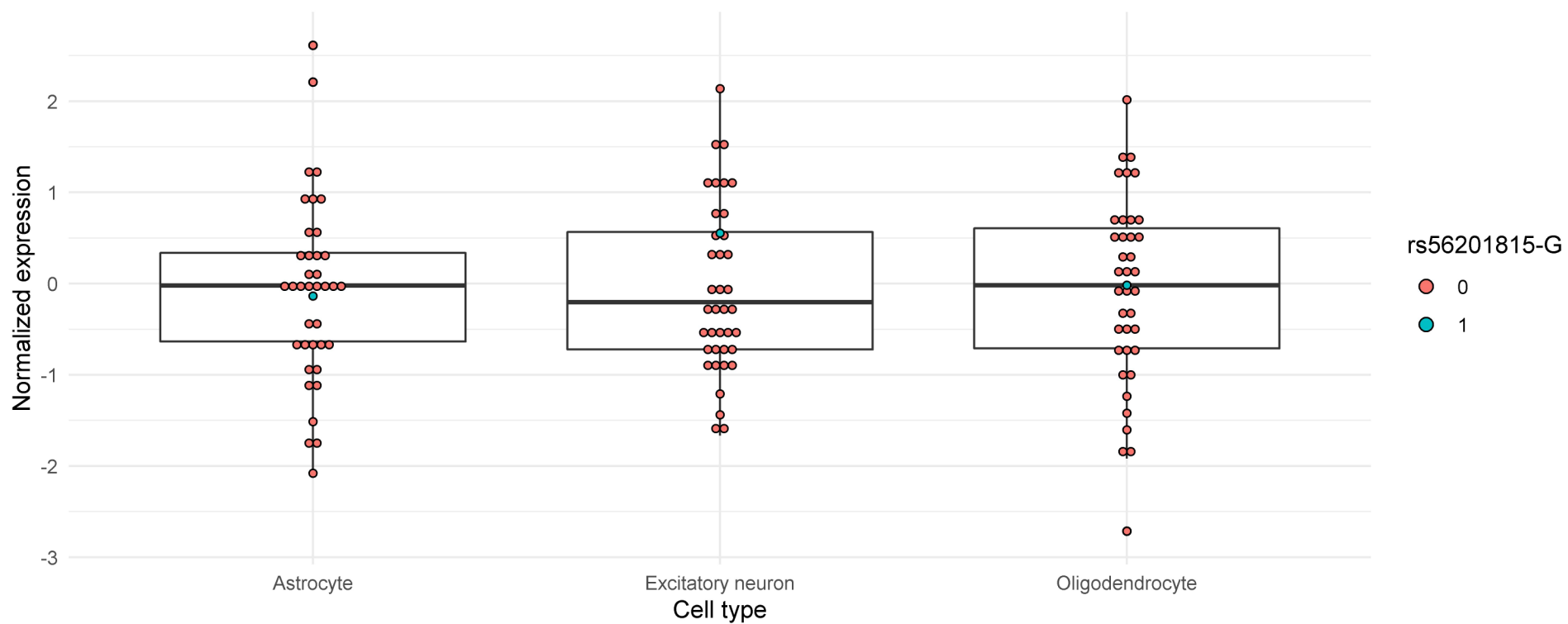

Supplement: Supplementary file 12 — Supplementary Figure S4 [file 41398_2021_1263_MOESM12_ESM.pdf]

A)

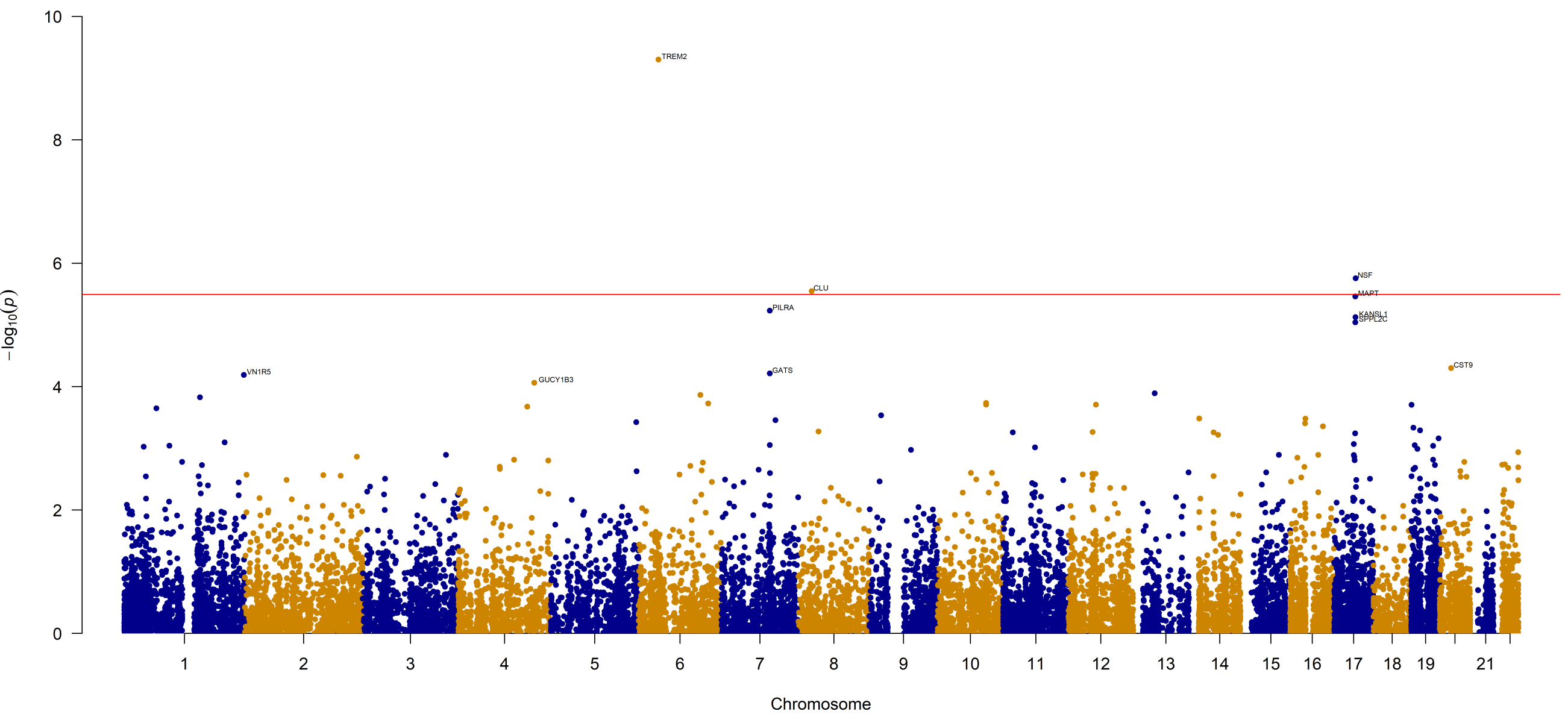

B)

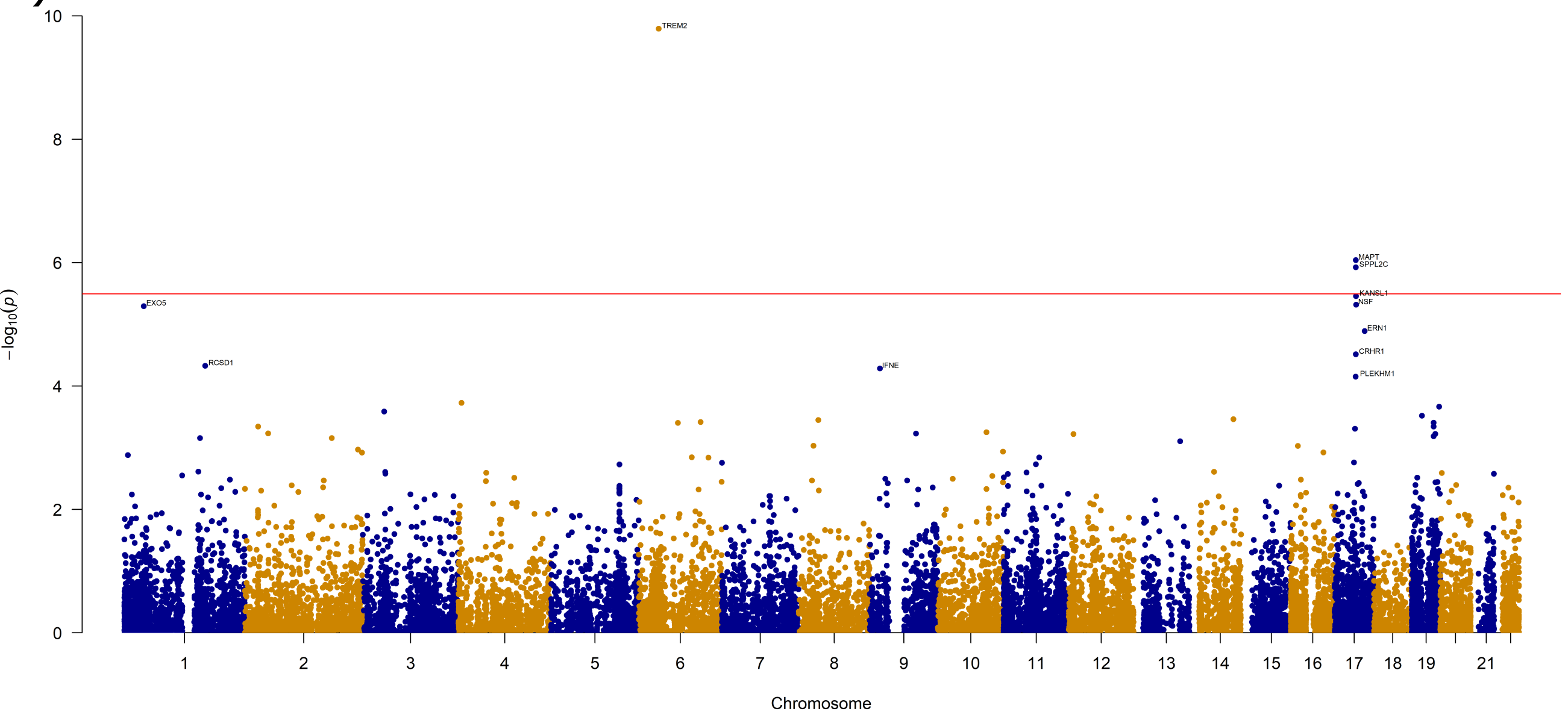

Supplement: Supplementary file 13 — Supplementary Figure S5 [file 41398_2021_1263_MOESM13_ESM.pdf]
